# Synthesis, Nanoformulations, and In Vitro Anticancer Activity of *N*-Substituted Side Chain Neocryptolepine Scaffolds

**DOI:** 10.3390/molecules27031024

**Published:** 2022-02-02

**Authors:** Ibrahim El-Tantawy El Sayed, Sami Ullah, Omar A. Al-Hartomy, Asmaa Mohammed Hasanein, Abdullah A. S. Ahmed, Khaled A. Kahilo, Mehrez E. El-Naggar

**Affiliations:** 1 Chemistry Department, Faculty of Science, Menoufia University, Shebin El Koom 32511, Egypt; ibrahimtantawy@yahoo.co.uk (I.E.-T.E.S.); asmaa_aboelnaga77@yahoo.com (A.M.H.); chemist_abdullah_2009@yahoo.com (A.A.S.A.); 2 Research Center for Advanced Materials Science (RCAMS), King Khalid University, P.O. Box 9004, Abha 61413, Saudi Arabia; samiali@kku.edu.sa; 3 Department of Chemistry, College of Science, King Khalid University, P.O. Box 9004, Abha 61413, Saudi Arabia; 4 Department of Physics, Faculty of Science, King Abdulaziz University, Jeddah 21589, Saudi Arabia; oalhartomy@kau.edu.sa; 5 Biochemistry Department, Faculty of Veterinary Medicine. Kafrelsheikh University, Kafr El Sheikh 3351, Egypt; kahilo2000@yahoo.com; 6 Institute of Textile Research and Technology, National Research Centre, Dokki, Giza 12622, Egypt

**Keywords:** nanoemulsion, anticancer activity, neocryptolepine scaffolds

## Abstract

The naturally occurring neocryptolepine (5-Methylindolo [2,3-*b*]quinoline) and its analogs exhibited prominent anticancer and antimalarial activity. However, the main problem of this class of compounds is their poor aqueous solubility, hampering their bioavailability and preventing their clinical development. To overcome the problem of insolubility and to improve the physicochemical and the pharmacological properties of 5-Methylindolo [2,3-*b*]quinoline compounds, this work was designed to encapsulate such efficient medical compounds into mesoporous silica oxide nanoemulsion (SiO_2_NPs). Thus, in this study, SiO_2_NPs was loaded with three different concentrations (0.2 g, 0.3, and 0.6 g) of **7b** (denoted as NPA). The findings illustrated that the nanoparticles were formed with a spherical shape and exhibited small size (less than 500 nm) using a high concentration of the synthesized chemical compound (NPA, 0.6 g) and good stabilization against agglomeration (more than −30 mv). In addition, NPA-loaded SiO_2_NPs had no phase separation as observed by our naked eyes even after 30 days. The findings also revealed that the fabricated SiO_2_NPs could sustain the release of NPA at two different pH levels, 4.5 and 7.4. Additionally, the cell viability of the produced nanoemulsion system loaded with different concentrations of NPA was greater than SiO_2_NPs without loading, affirming that NPA had a positive impact on increasing the safety and cell viability of the whole nanoemulsion. Based on these obtained promising data, it can be considered that the prepared NPA-loaded SiO_2_NPs seem to have the potential for use as an effective anticancer drug nanosystem.

## 1. Introduction

**Figure 1:**
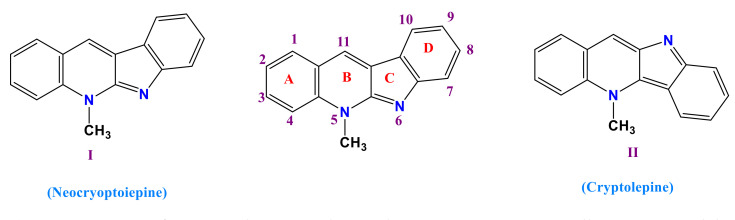
Structure of neocryptolepine I and cryptolepine II isomers as naturally occurring indoloquinoline alkaloids, including numbering.

Cancer is one of the most significant issues in the modern health system. In 2020, 19.3 million new cancer cases were registered, and 10 million people died from cancer [1]. Cancer is one of the leading causes of death globally, next to cardiovascular and infectious diseases. Therefore, the development of new, safer, and more efficient chemotherapeutics is of prime importance. The use of natural products as chemotherapeutic agents is well established; however, many of these agents are associated with undesirable side effects, including high toxicity and instability. Furthermore, the development of drug-resistant cancers makes the search for new anticancer lead compounds a priority [2]. In this context, extracts of the West African shrub *Cryptolepis sanguinolenta* were used for centuries against various disorders, amongst them malaria, in traditional African medicine [3]. The biologically active components of the plant extracts are indoloquinoline alkaloids such as neocryptolepine I and its regio-isomer, cryptolepine II (Figure 1). The alkaloid neocryptolepine I or 5-Methyl-indolo[2,3-b]quinoline has potent anticancer, antibacterial, antischistosomicidal, and antiplasmodial activity [4,5,6,7,8,9,10,11,12,13,14,15].

The anticancer activity of indoloquinolines is based on DNA binding, specifically by DNA base pair intercalation, followed by inhibition of the enzyme topoisomerase II [16]. Some indoloquinolines have shown a high affinity towards triplex and quadruplex DNA [17]. Furthermore, indoloquinolines such as neocryptolepine analogs show promising activity both as anticancer and antimalarial drugs. However, the main problem of this class of compounds is their poor aqueous solubility, hampering their bioavailability and preventing their clinical development. We hypothesize that the synthesis of nanoparticles based on neocryptolepine will lead to increased aqueous solubility and an improved pharmacological profile, since synergistic biological effects with nanoformulation are likely due to specific target delivery. The aim of this work is designed to improve the pharmacological profile of the parent natural alkaloid neocryptolepine via chemical modification through the installation of basic amino-substituted side-chains at the C-11 position of the parent compound. A basic side-chain proved to be an important feature for drug activity [2,8,9]. In addition, the aqueous solubility and pharmacological profile were improved by encapsulating neocryptolepine compounds into nanoparticles. Ultimately, this should lead to the development of novel compounds that are able to cure cancer and other diseases. Because of their distinctive morphologies, large surface areas, customizable pore architectures, highly modifiable surface characteristics, and high biocompatibility, silica nanoparticles have piqued interest in use in a variety of applications, including sensing, catalysis, and drug delivery [18,19].

The two most crucial properties to achieving the required medication concentration in the systemic circulation are solubility and controlled release of drug particles. A drug product requires transport drug particles to systemic circulation at an appropriate pace and extent to elicit the intended therapeutic response, which would be ultimately determined by the drug molecules’ bioavailability [20,21].

As a result, throughout the pharmaceutical development process, the poor bioavailability of novel chemical compounds is a key issue. Aqueous solubility, drug permeability, and dissolving rate are all variables that affect oral bioavailability. Bioavailability improvement of orally delivered drugs is achieved through a variety of methods. Nanoemulsions are one, and they appear to have received much attention because of their low toxicity and capacity to enhance drug bioavailability several times [22,23]. Nanoemulsions are isotropic mixtures of transparent or translucent oil globules distributed in an aqueous phase sustained by an interfacial coating of surfactant and cosurfactant molecules with a smaller droplet size that are kinetically stable [24,25]. By increasing solubility and shielding the active moiety from enzymatic degradation, they have the greatest promise for the oral administration of weakly water-soluble medicines [26]. Surfactants and cosurfactants are used in nanoemulsion formulation to change intestinal permeability, therefore enhancing drug absorption [27]. The ability of nanoemulsion to overcome inter and intrasubject differences in patients is one of its unique properties [28].

Herein, we introduce a facile, template-free method to synthesize uniformly sized silica oxide nanoemulsion loaded with the synthesized organic compound as a drug model. The as-prepared nanoemulsions were extensively characterized in terms of surface structure, particle shape, hydrodynamic particle size, and surface charge. The research work was extensively evaluated via determining the cytotoxicity, drug content, and in vitro release at two different pH^’^s.

## 2. Materials and Methods

### 2.1. Materials

Tetraethyl orthosilicate (purity more than 99%) was purchased from Aldrich Chemical Co., Inc., Milwaukee, WI, USA. Span 60 was purchased from Sigma-Aldrich Co. (Berlin, Germany). ^1^H-NMR and ^13^C-NMR experiments were investigated at Zagzig University using 400 MHz for ^1^H- NMR and 100 MHz for ^13^C-NMR Bruker. Chemical shifts relative to the solvent were measured in parts per million (ppm). FT-IR spectroscopy was recorded on Thermo Scientific at Faculty of Science, Menoufia University. Melting points (m.p.) were measured using the Stuart scientific melting point apparatus and are uncorrected. Without further purification, the solvents were utilized as obtained. Purity of 7a and 7b was verified using HPLC systems. HPLC was performed on Agilent 1100 integrated system equipped with a G1313A automated injector, a G1311A quaternary pump, and a G1315B diode-array detector (DAD). The chromatographic separation of the compounds was achieved with reversed-phase columns, ZORBAX XDB-C18 (5.0 × 150, 5 µm)) from Agilent (USA) operating at a constant flow rate of 1 mL/min and temperature (20 °C); water (A) and ACN (B), were used as eluents. The chromatographic data were analyzed using Agilent chemstation B.02.01. The starting materials were commercially available from Acros Organics as 1,3-diamino propane **6a** and 1,4-Bis(3-aminopropyl)-piperazine **6b**. The key starting 11-Chloroneocryptolepine **5** was synthesized as reported in the literature [7].

The general procedure for the synthesis of 11-aminoalkylamino neocryptolepine (7) was performed as follows: To 11-chloroneocryptolepine **5** (1 g, 3.75 mmol) was added in excess amount 1.3-diaminopropane 6a or 1.4-Bis(3-aminopropyl)-piperazine 6b (11.26 mmol) in DMF (1 mL) and (1.57 mL,11.26 mmol) of triethylamine as a base. The mixture was refluxed for 2 h. and cooled to room temperature, then poured into ice water; the precipitated product was filtered off, washed with water, and purified by recrystallization from methanol to yield a yellow solid of 7.

Synthesis of N1-(5-Methyl-5H-indolo[2,3-b]quinolin-11-yl)propane-1,3-diamine (**7a**)

Yellow solid yield (70%), m.p 71–73 °C, FT-IR (KBr) υ: 3216 cm^−1^ (NH), 2945 cm^−1^ (CH), 1584 cm^−1^ (C=C), 1205 cm^−1^ (C-C). ^1^H-NMR (CDCl_3_, 400 MHz, 7.26 ppm) ppm δ: 1.85 (t, 2H, CH_2_, *J* = 8 Hz), 3.06 (t, 2H, CH_2_*, J* = 8 Hz), 4.07 (m, 2H, CH_2_), 4.24 (s, 3H, *N*-CH_3_), 7.15–7.98 (m, 8H, CH_Ar_). ^13^C-NMR (CDCl_3_, 100 MHz) ppm δ: 29.1, 32.9, 41.5, 49.4, 114.7, 120.3, 121.4, 124.1, 125.7, 130.4, 136.1, 152.1. HPLC purity 95.89% (Figure 1S).

N-{3-[4-(3-Aminopropyl piperazin-1-yl)propyl]}-5-Methyl-5H-Indolo[2,3 b]quinolin-11-amine (7b).

Yellow solid, yield (65%) m.p 135–137 °C, FT-IR (KBr) υ: 3203 cm^−1^ (NH), 2938 cm^−1^ (CH), 1625 cm^−1^ (C=C), 1224 cm^−1^ (C-C). ^1^H-NMR (DMSO-d_6_ 400 MHz) ppm δ: 1.70 (m, 2H, CH_2_), 1.88 (m, 2H, CH_2_), 2.47–2.65 (m, 12H, 6CH_2_), 2.82 (m, 2H, CH_2_), 3.97 (m, 2H, CH_2_), 4.26 (s, 3H, *N*-CH_3_), 5.45 (brs, 2H, NH_2_), 7.13–8.24 (m, 8H, CH_Ar_), 8.61 (brs, 1H, NH). ^13^C-NMR (DMSO-d_6,_ 100 MHz) ppm δ: 24.0, 27.2, 33.7, 37.9, 47.2, 52.9, 55.3, 111.3, 115.7, 120.5, 122.8, 123.2, 126.2, 131.6, 135.2, 139.6, 147.3. in addition, the HPLC purity is 99.73%.

### 2.2. Preparation of NPA-Loaded SiO_2_ Nanoemulsion

Neocryptolepine analog (**7b**), denoted as NPA (0.2 g, 0.3 g, and 0.6 g), was dispersed in a solution containing 10 mL dimethyl sulfoxide (DMSO) and 2 mL of Span 60 as an emulsifying agent. The admixture was kept under continuous magnetic stirring for 15 min at room temperature. On the other hand, 30 mL of Tetraethyl orthosilicate (TEOS) was dissolved in a solution of deionized water (5 mL) containing 2 drops of span 60 and kept under stirring at room temperature for 15 min. At the end of dissolution and dispersion, NPA solution was added drop by drop to TEOS solution and subjected to mechanically stirring for another 3 min in order to affirm the encapsulation process of NPA inside the cavities of TEOS. After that, the ultrasonic process was used to further disperse the particles of the produced nanoemulsion. At this step, the whole solution appeared as a milky solution. After the preparation of SiO_2_ nanoemulsion encapsulated with 0.2 g, 0.3 g, and 0.6 g of NPA, the three obtained samples were labeled as SiO_2_@NPA-0.2, SiO_2_@NPA-0.3, and SiO_2_@NPA-0.6, respectively.

### 2.3. Characterization

The morphology of the prepared samples of silica oxide nanoparticles that were loaded with NPA, SiO_2_@NPA-0.2, SiO_2_@NPA-0.3, and SiO_2_@NPA-0.6, were investigated via the field emission scanning electron microscope technique using an accelerating voltage 30 KV (FE-SEM QUANTA FEG250, Republic of Czech). The elemental analysis, weight percent, and elemental mapping of the presented samples were identified using energy dispersive X-ray (EDX) analysis attached with the FE-SEM model (AMETA version). Before the investigation, the as-prepared nanoemulsions were submitted for the centrifugation process to precipitate the powders in wet form, followed by air drying.

A transmission electron microscope (TEM) was used to examine the particle shape of the synthesized compound-loaded SiO_2_ nanoemulsion using the TEM model (Hitachi H-7500, USA). Dynamic light scattering (DLS) was used to estimate the average hydrodynamic size and polydispersity index (PdI) of the prepared nanoemulsions. 5 mL of the prepared nanoemulsion was poured into Beckman Coulter Cuvettes, and measurements were taken to determine diameter size and PdI. The physical stability of the nanoemulsion was determined by particle charge. The zeta potential value, which is evaluated by electrophoretic mobility of particles in an electrical field, is used to quantify particle charge. The produced nanoemulsion’s zeta potential was determined using a Beckman Coulter DelsaTM Nanoparticle analyzer from the United States [29].

In Dulbecco’s Modified Eagle’s Medium, a human breast adenocarcinoma (MCF-7) cell line was maintained. 3-(4,5-dimethylthiazol-2-yl)-2,5-diphenyltetrazolium bromide (MTT) was used for the cell viability experiments.

By dissolving 1 mL of the nanoemulsion in 10 mL of methanol and shaking the mixture for 30 min in an incubator, the drug concentration was measured. After 15 min, the supernatant was collected and analyzed with a UV spectrophotometer (UV-1700 Pharma Spec, Shimadzu, Japan) at 278 nm to calculate the drug concentration.

The formulations of SiO_2_ nanoemulsion loaded with different concentrations of NPA were tested in vitro using phosphate buffer solution (PBS) at pH 5.5 and 7.4 with the utilization of dialysis membrane. The membrane (pore size: 12 KD) was triggered by soaking it in PBS overnight. The glycerin-based ingredients were removed by exposing it to running water for 3 h. The nanoemulsion formulations were placed into the dialysis membrane immersed in PBS (100 mL) and submitted for shaking at 50 rpm and 37 °C using a shaking incubator. At predefined intervals, samples were removed and replaced with an equal volume of fresh solution. After that, the in vitro release for each sample was determined using a UV/Visible spectrophotometer at 278 nm.

## 3. Results and Discussion

The goal of this study was to investigate the possibility of silica oxide nanoemulsion for augmenting NPA bioavailability. Nanoemulsion is one of the most widely utilized methods for overcoming drug solubility and bioavailability issues. Many pharmaceutical drugs benefit from nanoemulsions because they improve transparency, bioavailability, and shelf life. Nanoemulsion can be formed from an admixture of oil, water, surfactant, and cosurfactant [25,30]. Most of the prepared nanoemulsions are clear or translucent liquids with very small droplets, low interfacial tension, and long-term physical stability. Nanoemulsions have the potential to improve the dispensability of insoluble drugs [31,32]. Nanoemulsions provide a variety of advantages, including high drug solubility, good thermodynamic stability, and ease of manufacture [23,33].

As a result, nanoemulsions were designed and developed with the goal of increasing the NPA needed bioavailability. Because of its compliance with the production procedure and expected benefits in overcoming bioavailability concerns, nanoemulsion was selected as the best solution for improving the bioavailability of NPA drugs. By increasing the solubility and permeability of NPA, issues with inadequate bioavailability can be relieved. The ultrasonication process was used to create nanoemulsion formulations that were optimized in terms of shape, particle size and dispersion, zeta potential, cytotoxicity, drug content, and in vitro NPA release.

### 3.1. Synthesis of 11-Chloroneocryptolepine **5**

**Scheme 1:**
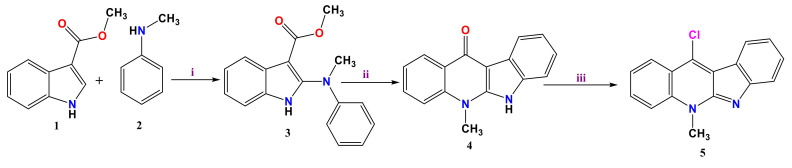
Synthesis of the neocryptolepine **5**.Reagents and conditions:(i) a. *N*-chlorosuccinimide, 1,4-dimethylpiperazine, CH_2_Cl_2_, 0 ºC, 2b. trichloroacetic acid, room temperature, 2 h (ii) diphenyl ether, reflux, 3 h (iii) POCl_3_, toluene, reflux, 12 h.

The pathway to assemble the neocryptolepine core has been developed starting from easily accessible intermediates, as depicted in Scheme 1. This approach allowed us to synthesize new analogs with a varied substitution pattern at the B ring. The synthetic methodology for 11-chloroneocryptolepine **5** was achieved starting from 1H-methyl indole-3-carboxylate **1** and *N*-methylaniline **2**. The intermediate Methyl 2-(methyl(phenyl)amino)-1H-indole-3-carboxylate **3** was obtained via chlorination with *N*-chlorosuccinimide (NCS) in the presence of 1,4-dimethylpiperazine, followed by the addition of *N*-methylaniline **2** as trichloroacetate salt. The resulting intermediate was cyclized in boiling diphenylether to afford **4**. Dehydroxychlorination with POCl_3_ then give 11-chloroneocryptolepine **5** (Scheme 1).

### 3.2. Formation of Amino Neocryptolepine **7**

**Scheme 2:**
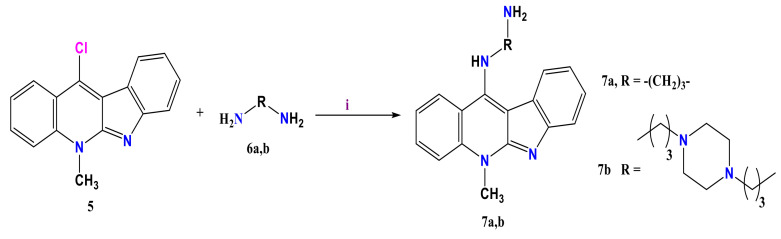
Synthesis of 11-aminoalkylamio neocryptolepine free amine. Conditions: (i) 3 times excess, DMF, reflux, 2 h.

11-Chloroneocryptolepine **5** was condensed with diamine derivatives **6a,b** in the presence of few drops of DMF to afford the corresponding 11-aminoalkylaminoneocryptolepine derivatives **7a** & **7b** via nucleophilic aromatic substitution (SN_Ar_) reaction mechanism, in which the substitution of the amino group to chlorine atom of **5** at the unsaturated sp^2^ C-11 position takes place as depicted in Scheme 2. This reaction proceeds through the addition of the amino group (: Nu–) to form a resonance-stabilized anion with a new C–N bond followed by elimination of HCl as triethyl amine hydrochloride salt to afford the end products **7a** and **7b** as depicted in (Scheme 2).

The synthesized 11-amino alkyl amino neocryptolepine free amines **7** was elucidated throughout different spectroscopic methods as FT-IR and NMR. The FT-IR spectrum showed the absorption bands of the (NH) functional group at 3216 and 3203 cm^−1^ for **7a** and **7b**, respectively. On the other hand, the ^1^H-NMR spectrum showed the characteristic (*N*-CH_3_) of neocryptolepine core reported at 4.24 ppm for **7a** and 4.26 ppm for **7b**. Meanwhile, in ^13^C-NMR the (*N*-CH_3_) group was detected at 41.56 ppm for **7a** and 39.36 ppm for **7b**.

### 3.3. Characterization of the As-Synthesized NPA (**7b**)-Loaded SiO_2_ Nanoemulsion

**Figure 2:**
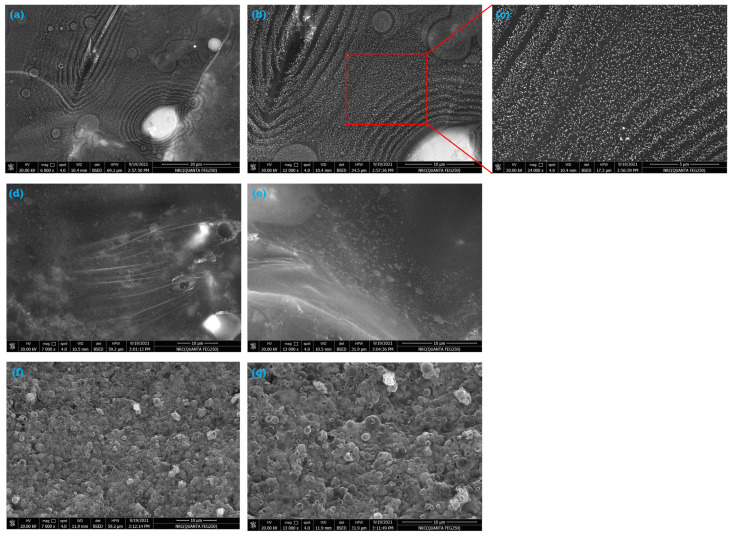
SEM of (**a**–**c**) SiO_2_@NPA-0.2, (**d**,**e**) SiO_2_@NPA-0.3 and (**f**,**g**) SiO_2_@NPA-0.6.

The topographical surface at different magnifications of the prepared nanoemulsion based on SiO_2_@NPA-0.2, SiO_2_@NPA-0.3, and SiO_2_@NPA-0.6 is demonstrated in Figure 1. As shown in Figure 1a–c, SiO_2_@NPA-0.2 exhibit homogenous particles with very small sizes and ordered shapes, implying that SiO_2_ nanoparticles have a definite porous structure, and these pores are completely encapsulated with NPA with no chance for NPA to leach out the cavity of SiO_2_ nanoparticles. In addition, this phenomenon proves that in vitro release of NPA from SiO_2_ porous structure will be sustained during drug delivery domains. Additionally, increasing the concentration of the loaded NPA to 0.3 g (SiO_2_@NPA-0.3) (Figure 1d,e) leads to the formation of nanoemulsion with good particle distribution, however, with the appearance of small particles agglomeration. The surface structure of the nanoemulsion is completely changed while increasing the concentration of NPA to 0.6 g, as shown in Figure 2f,g.

**Figure 3:**
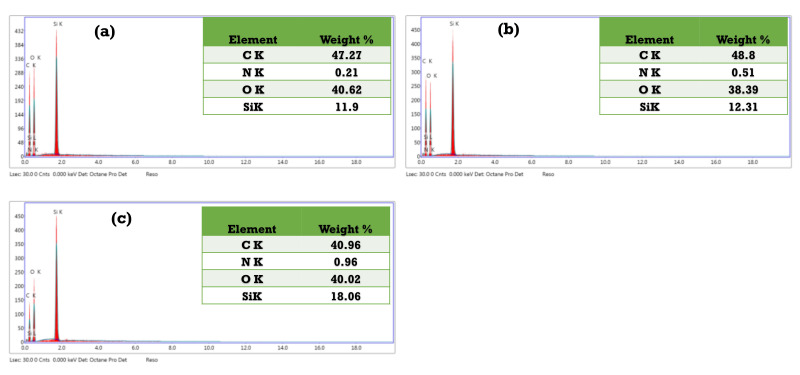
EDX and elemental analysis of (**a**) SiO_2_@NPA-0.2, (**b**) SiO_2_@NPA-0.3, and (**c**) SiO_2_@NPA-0.6.

For further confirmation, EDX was used to identify the elemental analysis of the prepared samples, and the data were set in Figure 3. It is observed that the samples SiO_2_@NPA-0.2 (Figure 3a), SiO_2_@NPA-0.3 (Figure 3b), and SiO_2_@NPA-0.6 (Figure 3c) are comprised of C, N, O, and Si. Each onset table contains the weight percent of each element.

The presence of C and O can be attributed to the presence of the encapsulated organic compound (NPA, 7b). In addition, the presence of N also confirmed the presence of NPA that loaded SiO_2_NPs. There is a peak for Si, which also affirmed the presence of SiO_2_NPs. From these figures, it could be certain that NPA is successfully encapsulated into the utilized SiO_2_NPs.

It can also be seen that with increasing the concentration of NPA-loaded SiO_2_ nanoparticles, the weight percent of nitrogen is increased due to the loading capacity of NPS into and onto the prepared nanoparticles. By and large, the weight percent of nitrogen for the three prepared samples, SiO_2_@NPA-0.2, SiO_2_@NPA-0.3, and SiO_2_@NPA-0.6, is 0.21, 0.51, and 0.96%, respectively.

**Figure 4:**
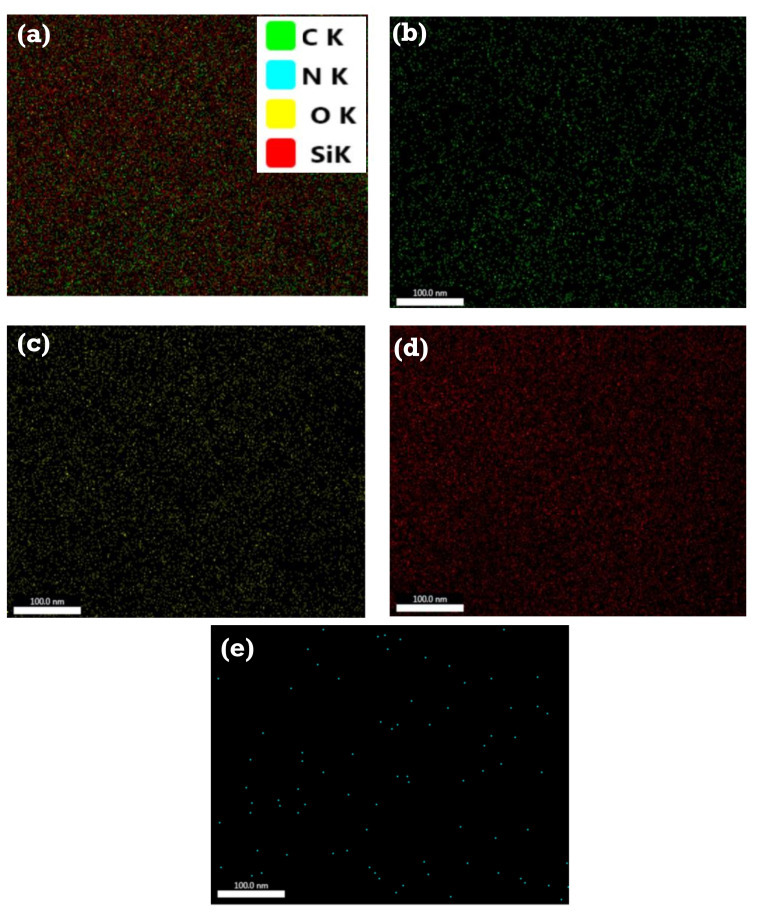
Elemental mapping of SiO_2_@NPA-0.6: (**a**) all elements, (**b**) carbon element, (**c**) oxygen element, (**d**) Si element and (**e**) nitrogen element.

The sample (SiO_2_@NPA-0.6) was selected to investigate the mapping and distribution of each element (Figure 4). Thus, Figure 4 shows the elemental mapping of the characterized sample. Figure 4a illustrates the mapping of all elements. Each element is marked with a different color. Figure 4b–e demonstrated the mapping of C, O, Si, and N, respectively. The mapping images are in agreement with EDX peaks and the elements’ weight percent.

**Figure 5:**
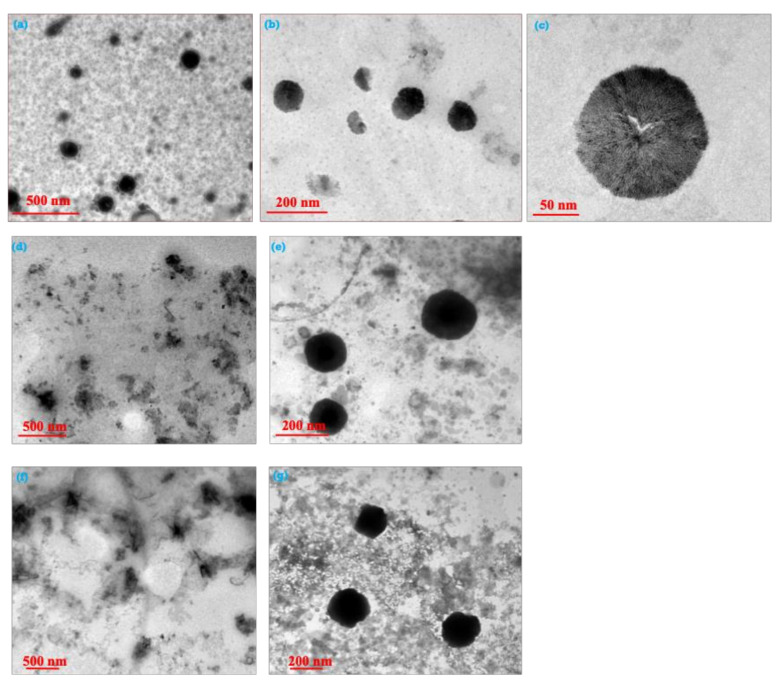
TEM of (**a**–**c**) SiO_2_@NPA-0.2, (**d**,**e**) SiO_2_@NPA-0.3, and (**f**,**g**) SiO_2_@NPA-0.6.

For further illustration, TEM was utilized to investigate the particle shape of the as-prepared SiO_2_ nanoemulsion loaded with different concentrations of NPA. As determined, TEM images for the analyzed samples were taken at different magnifications. Thus, Figure 5a–c outlines TEM at three different magnifications of SiO_2_ nanoemulsion loaded with 0.2 g of NPA. It can be discerned that the cavity of the nanosized SiO_2_ encapsulated with NPA with no noticeable leaching for NPA, affirming that SiO_2_ nanoemulsion is capable of encapsulating the added NPA (0.2 g) and leads to the formation of spherical size with good dispersion. Meanwhile, Figure 5d,e displays TEM of SiO_2_ nanoemulsion loaded with 0.3 g of NPA. It is observed that the particles are still small in size but with significant agglomeration. On the other hand, by increasing the loaded concentration of NPA to 0.6 g (Figure 5f,g), the nanoemulsion of SiO_2_ is completely encapsulated with some of the added NPA. The other particles of NPA are leached away from the core of SiO_2_ nanoemulsion, which leads to the formation of deep black colors and noticeable agglomeration. The particles of NPA-loaded SiO_2_ (0.6 g) nanoemulsion are formed with large particles due to the agglomeration.

It can be concluded that beyond the spherically packed pore structure, TEM images reveal a layer of material deposited on the exterior of these particles. This exterior substance is thought to be drug crystallized on the surface of particles. In order to determine the average hydrodynamic size, polydispersity index (PdI), and zeta potential of SiO_2_ nanoemulsion encapsulated with different concentrations of NPA (0.2 g, 0.3 g, and 0.6 g), dynamic light scatterings (DLS) were assessed. The particle size and size distribution (PdI) of nanoemulsions are significant in predicting the drug and carrier physical stability and in vivo rate. The lack of flocculation is due to the small particle size, which allows the system to stay dispersion without any separation.

**Figure 6:**
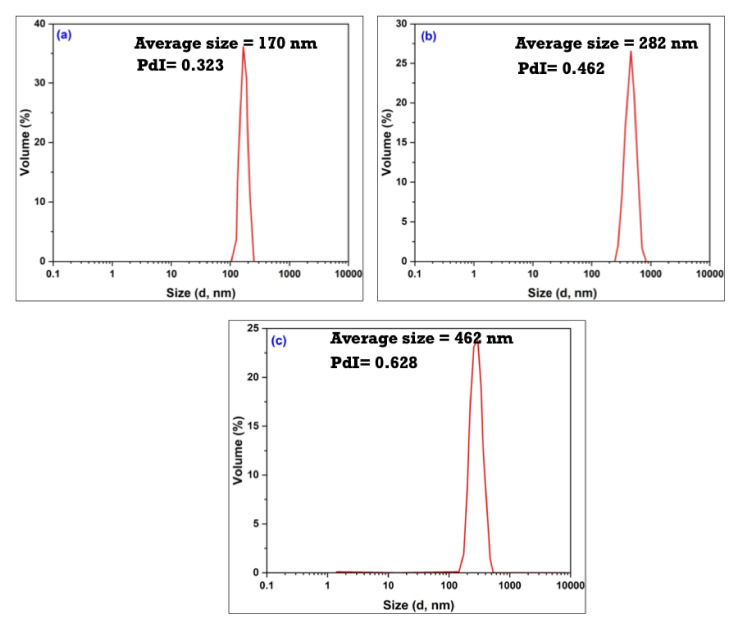
Average hydrodynamic size and polydispersity of (**a**) SiO_2_@NPA-0.2, (**b**) SiO_2_@NPA-0.3, and (**c**) SiO_2_@NPA-0.6.

As shown in Figure 6, the average particle size increases with increasing the concentration of the loaded NPA. At a low concentration of NPA (0.2 g), as shown in Figure 6a, the particle size is 170 nm with PdI equal to 0.323. The size of SiO_2_@NPA-0.2 is small with monodispersity due to the low value of PdI, which is less than 0.5. As well established, PdI with values between 0 and 0.5 is shown for the formed particles with homogeneity and monodispersity. Meanwhile, PdI values more than 0.5 to 1 confirmed that the produced particles have heterogeneous shapes [34]. Thus, Figure 6b depicts the diameter of SiO_2_@NPA-0.3 increases to 282 nm with PdI reaching 0.462, while SiO2@NPA-0.6 (Figure 6c) gives size with a significantly increased particle diameter (462 nm) and PdI (0.628). For these data, it can be concluded that the average particle size obtained from DLS is in accordance with TEM images.

Moving to zeta potential assessment that gives information about the stability of the prepared nanoemulsion against dispersion or agglomeration during storage [35,36]. The surface charge of the particles is represented by the zeta potential, which indicates the degree of repulsion between the charged-like particles surrounding the neighboring particle. The interaction forces amongst particles at the nanoemulsion’s surfaces are referred to as zeta potential values, and they influence the nanoemulsion’s stabilization [37].

Initially, by the naked eye, it can be observed that the prepared nanoemulsions are formed as one phase with no remarkable separation.

In order to confirm this step of the absence of phase separation, zeta potential (surface charge) for the prepared nanoemulsion samples was conducted. As known, the zeta potential value above −30 mv depicts the good stability of the prepared particles [38]. Meanwhile, the zeta potential value less than −30 mv illustrates that the particles are formed with poor stability.

**Figure 7:**
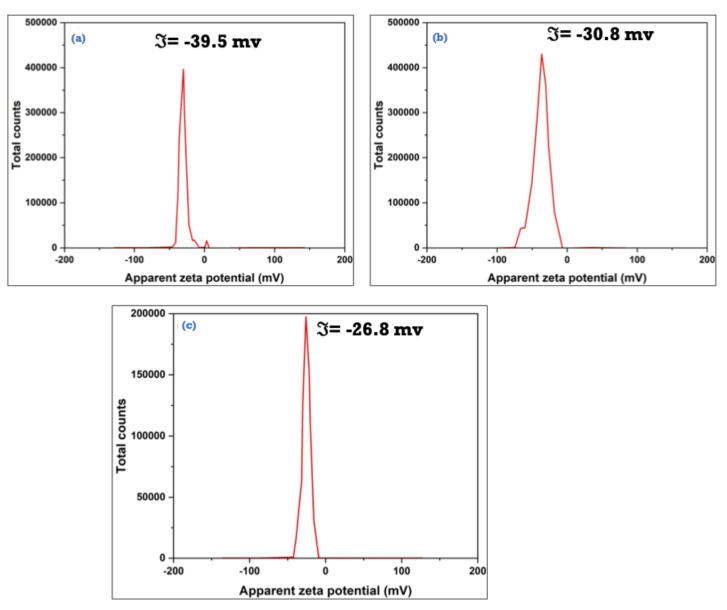
Zeta potential of (**a**) SiO_2_@NPA-0.2, (**b**) SiO_2_@NPA-0.3 and (**c**) SiO_2_@NPA-0.6.

Thus, Figure 7a–c displays the zeta potential values for the nanoemulsion samples SiO_2_@NPA-0.2, SiO_2_@NPA-0.3, and SiO_2_@NPA-0.6 reach −39.5 mv, −30.8 mv, and −26.8 mv, respectively. Following the rule of zeta potential, it is depicted that SiO2@NPA-0.2 is more stable than SiO_2_@NPA-0.3 and SiO2@NPA-0.6. Thus, it is affirmed from obtained zeta potential values that the data is in accordance with that of TEM and DLS.

### 3.4. Cytotoxicity of the Prepared Nanoemulsions Loaded with NPA (7b) As Drug Model

**Figure 8:**
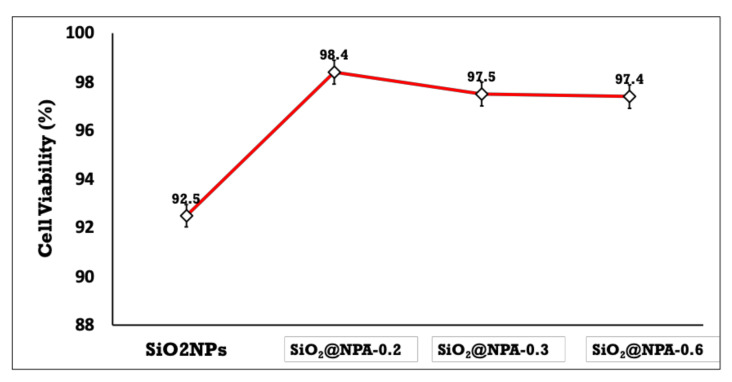
Cytotoxicity of the prepared nanoemulsions based on SiO_2_NPs, SiO_2_@NPA-0.2, SiO_2_@NPA-0.3, and SiO_2_@NPA-0.6.

Figure 8 presents the in vitro cytotoxicity of SiO_2_NPs, SiO_2_@NPA-0.2, SiO_2_@NPA-0.3, and SiO_2_@NPA-0.6 with cancer cell nominated MCF-7). Initially, when SiO_2_NPs was applied at a concentration of 500 μg mL^−1^ to the cancer cell (MCF-7), there was no significant difference in cancer cell death. According to these findings, SiO_2_NPs is biocompatible and has no toxic effect.

On the other hand, when the sample containing the drug (SiO_2_@NPA-0.2, SiO_2_@NPA-0.3, and SiO_2_@NPA-0.6) were applied at the same concentration; the extent of cancer cell death was more pronounced, implying that each SiO_2_ nanoemulsion and the nanoemulsion loaded with different concentrations of NPA had with excellent performance and positive impact against carcinogenic cells.

### 3.5. Drug Content and In Vitro Release of NPA at Two Different pH’s

For the prepared samples of SiO_2_@NPA-0.2, SiO_2_@NPA-0.3, and SiO_2_@NPA-0.6, the drug content of NPA-loaded nanoemulsion was determined to be 97.33 ± 0.75%, 98.32 ± 0.65%, and 98.76 ± 0.45%, respectively. The nanoemulsion had a strong drug loading capacity, which was an important prerequisite for drug delivery and in vitro release.

**Figure 9:**
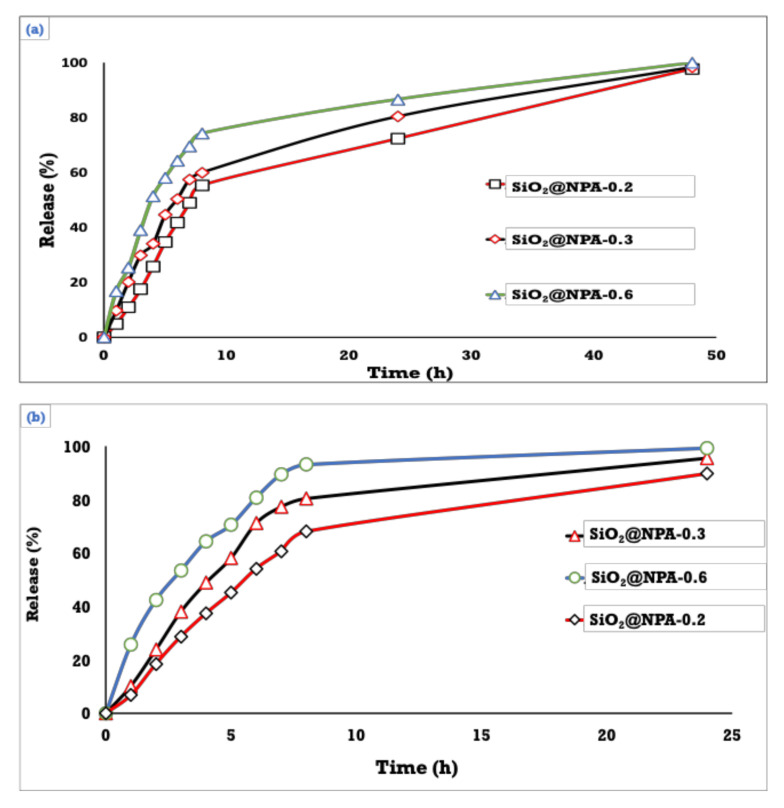
The release profile of NPA from the designed nanoemulsion system at two different pH’s (**a**) pH 7.4 (**b**) 5.5.

In vitro drug release of NPA from the prepared SiO_2_ nanoemulsion was carried out using phosphate buffer saline (PBS). Figure 9a,b illustrates in vitro release of NPA at two different PH’s; 5.5 and 7.3, respectively. As shown before, in vitro release of the three samples coded with SiO_2_@NPA-0.2, SiO_2_@NPA-0.3, and SiO_2_@NPA-0.6 were studied at different times lengths, from 0 to 50 h. As illustrated in Figure 8a,b, all samples undergo two phases—fast release followed by slow release (controlled release). The fast release at the initial time can be ascribed to the amount of NPA that is physically adsorbed onto the surface of SiO_2_ nanoparticles. Meanwhile, the slow release can be attributed to the well-encapsulated NPA with the prepared SiO_2_ nanoparticles. The in vitro release of NPA at pH 5.5 from the nanoemulsion system SiO_2_@NPA-0.2, SiO_2_@NPA-0.3, and SiO_2_@NPA-0.6 reaches 57.4, 60.2, and 74.3%, respectively, after 24 h. Meanwhile, for the same samples, the in vitro release at pH 7.4 reaches 63.4, 66.7, and 79.4%. It is observed that the in vitro release of NPA at pH 7.4 is more sustained compared with the release at pH 5.5. It can also be demonstrated that the in vitro release of NPA from SiO_2_@NPA-0.2 is more controlled than released from SiO_2_@NPA-0.3 and SiO_2_@NPA-0.6. Overall, at pH 5.5 and 7.4, the in vitro release of NPA continued for approximately 24 h and 50 h, respectively. From this observed data, it is implied that NPA is more favorable to be used for medical purposes at pH 7.4 rather than 5.5.

By and large, owing to its crystalline nature, lipophilicity, and low solubility in water, NPA showed sustained behavior even at two different pH levels due to the potential of SiO_2_ nanoparticles to completely entrap most of the loaded NPA into the small cavities of nanoparticles, which led to decreased dissolution rate and the drug release. It is also because of the tiny droplet size, which provides a considerable surface area for drug NPA release. As a result, nanoemulsion offered a far superior release profile.

## 4. Conclusions

The effective and efficient encapsulation of amino-substituted neocryptolepine scaffolds into mesoporous silica oxide nanoemulsion with high stability was demonstrated. The use of nanoformulation with the natural-based neocryptolepine analogs improved their effectiveness in the reduction of cancer cell viability and increased selectivity. Our initial goal was to prepare amino-substituted neocryptolepine incorporated into mesoporous silica oxide nanoemulsion to achieve the potential applications in the delivery of this class of natural-based compounds with good solubility and bioavailability with higher anticancer activity as well as lower cytotoxicity. Further, in vivo study is needed to validate our hypothesis. Due to their relatively simple methods of preparation, control over particle size and shape, high drug loading, and controlled drug delivery, silica oxide nanoparticles are promising drug nanocarriers. Mesoporous silica oxide nanoparticles have been prepared using tetraethyl orthosilicate (TEOS) and span 60 as alkoxide precursor and surfactant, respectively. The particle form changes from distributed nanospheres to agglomerates when the NPA concentration changes, as does the hydrolysis of TEOS and the micellization of span 60. As shown by TEM images, the addition of span 60 causes the formation of a silica mesoporous structure. The as-prepared silica oxide nanoparticles were fully investigated in terms of particle shape, particle size, and zeta potential. It can be concluded from these characterizations that silica oxide nanoparticles loaded with different concentrations of NPA exhibited spherical shape with good stability. The findings reveal that NPA can be loaded efficiently into mesoporous silica nanoparticles. The loading process has an impact on the loading extent. The amount of NPA molecules entrapped increased dramatically after several impregnations of mesoporous silica oxide nanoparticles in a solution of NPA in a DMSO solution. The cytotoxicity data demonstrated that the nanoparticles loaded with the synthesized anticancer drug did not have any noticeable toxicity when evaluated against human cell lines. These suggest further preclinical investigations to explore their potential applications in drug delivery.

## Data Availability

Not applicable.
